# Suppressive Regulation of KSHV RTA with *O*-GlcNAcylation

**DOI:** 10.1186/1423-0127-19-12

**Published:** 2012-02-02

**Authors:** Ying-Chieh Ko, Wan-Hua Tsai, Pei-Wen Wang, I-Lin Wu, Shu-Yu Lin, Yu-Lian Chen, Jen-Yang Chen, Su-Fang Lin

**Affiliations:** 1 National Institute of Cancer Research, National Health Research Institutes, Miaoli County, Taiwan; 2 NRPGM Core Facilities for Proteomics and Glycomics, Institute of Biological Chemistry, Academia Sinica, Taipei, Taiwan

**Keywords:** KSHV, K-RTA, *O*-GlcNAcylation, PARP1, Polycomb group (PcG) complex

## Abstract

**Background:**

The replication and transcription activator (RTA) of Kaposi's sarcoma-associated herpesvirus (KSHV) is a molecular switch that initiates a productive replication of latent KSHV genomes. KSHV RTA (K-RTA) is composed of 691 amino acids with high Ser and Thr content (17.7%), but to what extent these Ser and Thr are modified *in vivo *has not been explored.

**Methods:**

By using tandem mass spectrometric analysis of affinity-purified FLAG tagged K-RTA, we sought to identify Ser and Thr residues that are post-translationally modified in K-RTA.

**Results:**

We found that K-RTA is an *O*-GlcNAcylated protein and Thr-366/Thr-367 is the primary motif with *O*-GlcNAcylation *in vivo*. The biological significance of *O*-GlcNAc modified Thr-366 and Thr-367 was assessed by site-specific amino acid substitution. Replacement of Thr with Ala at amino acid 366 or 367 caused a modest enhancement of K-RTA transactivation activity in a luciferase reporter assay and a cell model for KSHV reactivation. By using co-immunoprecipitation coupled with western blot analysis, we showed that the capacity of K-RTA in associating with endogenous PARP1 was significantly reduced in the Thr-366/Thr-367 *O*-GlcNAc mutants. PARP1 is a documented negative regulator of K-RTA that can be ascribed by the attachment of large negatively charged polymer onto K-RTA via PARP1's poly (ADP-ribose) polymerase activity. In agreement, shRNA-mediated depletion of *O*-GlcNAc transferase (OGT) in KSHV infected cells augmented viral reactivation and virus production that was accompanied by diminished K-RTA and PARP1 complexes.

**Conclusions:**

KSHV latent-lytic switch K-RTA is modified by cellular *O*-GlcNAcylation, which imposes a negative effect on K-RTA transactivation activity. This inhibitory effect involves OGT and PARP1, two nutritional sensors recently emerging as chromatin modifiers. Thus, we speculate that the activity of K-RTA on its target genes is continuously checked and modulated by OGT and PARP1 in response to cellular metabolic state.

## Background

The replication and transcription activator K-RTA (also known as ORF50 or Lyta) is the immediate-early protein of Kaposi's sarcoma-associated herpesvirus (KSHV) that orchestrates and completes a KSHV lytic cycle of replication in many cell backgrounds. Genetic knockout of K-RTA resulted in a null phenotype in viral DNA synthesis and in virus production [1], emphasizing the essential role of K-RTA in the course of KSHV latent-lytic conversion. K-RTA interacts with and is regulated by a variety of host factors for its full functionality. Specifically, mutations or deletions introduced in the responsive elements present in the viral genome of K-RTA's interacting partners Oct-1 [2], RBPJκ [3] or C/EBPα [4] impaired K-RTA-mediated viral gene expression, suggesting these molecules are positive regulators of K-RTA. By contrast, interactions with host factors hKFC, PARP1 [5], K-RBP [6] or TLE2 [7] were reported to reduce K-RTA biological activities, indicating these molecules are negative regulators. How these regulators work in concert to determine the activity of K-RTA, and ultimately the fate of KSHV infection is of great interest in this field.

First discovered by Hart and colleagues in 1984 [8], *O*-GlcNAcylation is one of the most common post-translational modifications existing in numerous nucleoplasmic proteins. Distinct from *N*-linked glycosylations, which are found frequently in elongated forms attaching to extracellular glycoproteins, *O*-GlcNAcylation involves the addition of a single *N*-acetylglucosamine moiety onto the hydroxyl group of Ser or Thr residues, formally known as *O-*linked β-*N*-acetylglucosamine (*O*-GlcNAc). *O*-GlcNAcylation is a dynamic process that is catalyzed by *O*-GlcNAc transferase (OGT) and reversed by *O*-GlcNAcase (also known as OGA, NCOAT, MGEA5) [9]. Because both *O*-GlcNAcylation and *O*-phosphorylation act on the side chains of Ser and Thr residues, interplays between the two reactions have long been suspected. It has now been confirmed that crosstalk between *O*-GlcNAcylation and *O*-phosphorylation is not only active but also multifaceted. First, the turnover rates of *O*-GlcNAc and *O*-phosphate are very similar [10]. Second, OGT coexists with protein phosphatase 1β/γ in a functional complex [11]. Third, large-scale proteomic analysis revealed that the crosstalk between *O*-GlcNAcylation and *O*-phosphorylation can be derived from direct competition for a structural occupancy or by alteration of each other's enzyme activity via reciprocal modifications [12].

*O*-GlcNAcylation also plays a crucial role in transcriptional regulation. First of all, a great number of factors in the transcription machinery, including RNA polymerase II and transcription factors, are modified by *O*-GlcNAc [9]. In addition, OGT is known to tightly associate with the mSIN3A-HDAC complex [13]. Recently, *Drosophila *OGT was found to be a product of Polycomb group gene, *sxc*, and was involved in epigenetic gene silencing [14,15]. Furthermore, Myers et al. showed that a correct cellular level of *O*-GlcNAc was required for a proper transcriptional repertoire in mouse embryonic stem cells during early development [16]. It bears to note that *O*-GlcNAcylation-mediated transcriptional regulation can convey either a positive or a negative effect depending on the promoter context and cell system.

PARP1 is a transferase that adds to proteins the negatively charged polymer ADP-ribose (PAR) from NAD^+^. In response to DNA strand breaks, the catalytic activity of PARP1 is rapidly stimulated and robustly PARylates itself and various target proteins including core and linker histones, DNA-PK, topoisomerase I and transcription factors. Because the size and large negative charge of PAR, the addition of PAR may alter the function of target proteins, including enzymatic activity and protein-DNA interactions. For example, auto-PARylated PARP1 loses its affinity with DNA and dissociates from the chromosome, which allows the access of other DNA repair enzymes to the injured lesions [17,18]. In addition to DNA breaks, PARP1 also binds to special DNA structures including crossovers, cruciforms and supercoils. With this regard, PARP1 binds to KSHV terminal repeat/*Ori*P and is linked to viral latent genome maintenance [19].

Previously, by mass spectrometric analysis of affinity-purified K-RTA proteins, we identified two major modification sites on K-RTA: one was located at Thr-513 and Thr-514 that involved protein phosphorylation [20] and the other one was located at Thr-366 and Thr-367 that was *O*-GlcNAc modified. Here, we began with focusing on the role of *O*-GlcNAcylated Thr-366 and Thr-367 in K-RTA activity. We observed that *O*-GlcNAcylation state of K-RTA was reversely correlated with its transactivation activity. This inhibition might result from the recruitment of PARP1 by *O*-GlcNAcylated K-RTA. We also noticed that the level of cellular OGT was reversely related to the amplitude of KSHV lytic cycle reactivation. Thus, our findings suggest that host utilizes *O*-GlcNAcylation to counteract and dampen KSHV reactivation.

## Methods

### Reagents and Antibodies

D-(+)-glucosamine hydrochloride (G1514; Sigma), *N*-acetyl-D-glucosamine (GlcNAc, A8625, Sigma), O-(2-acetamido-2-deoxy-D-glucopyranosylidene)amino-*N*-phenylcarbamate (PUGNAc, A157250, Toronto Research Chemicals). Antibodies used in this study were M2-FLAG (F1804, Sigma), β-actin (A5441, Sigma), OGT (sc-74546, Santa Cruz Biotechnology, Inc.), PARP1 (sc-7150, Santa Cruz), GAPDH (#54593, AnaSpec, Inc.), α-tubulin (Millipore), RL2 (MA1-072, Affinity BioReagents), CTD110.6 (MMS-248R, Covance). Mouse monoclonal antibody of K-RTA was obtained from Dr. Keiji Ueda at Osaka University Graduate School of Medicine, Japan. Rabbit polyclonal antibody of K-RTA was obtained from Dr. Yoshihiro Izumiya at University of California, Davis. Rabbit polyclonal antibody of K-bZIP was obtained from Dr. Mengtao Li at School of Dentistry, University of California, Los Angeles.

### Plasmids

pLenti4-Flag-CPO and pLenti4-Flag-Luc were kindly provided by Dr. Dan Robinson at MCTP, University of Michigan (Ann Arbor, MI). pLenti4-Flag-CPO is a modified vector derived from pLenti4/TO/V5-DEST (Invitrogen). Briefly, the original *att*R1 site to V5 epitope region (nt 2405-4203) in pLenti4/TO/V5-DEST was replaced with an in-frame DNA fragment encoding Kozak sequence, ATG, FLAG tag and a rare cutter CPO I site (5'CGGTCCG). pLenti4-Flag-KRTA was constructed by cloning the coding sequences of K-RTA (GeneID: 4961526; Genebank: U71367.1) into the CPO I site of pLenti4-Flag-CPO. The expression plasmids for T366A, T367A and T366A/T367A were derived from pLenti4-Flag-KRTA by using QuikChange^® ^site-directed mutagenesis kit (Stratagene) with appropriate primers. In luciferase reporter assays, the upstream sequences of PAN (nt 28159 to 28660 of U75698) and ORF57 (nt 81556 to 82005) were cloned into Sac I-Xho I sites of pGL3-Basic (Promega), yielding pGL3-Basic-PANp and pGL3-Basic-ORF57p, respectively.

### Establishment of 293TetKR cells

T-REx™ HEK293 cells (Invitrogen) were infected with lentivirions harboring pLenti4-Flag-KRTA by using ViraPower™ system (Invitrogen). Forty-eight h after infection, the cells were selected with 400 μg/ml zeocin (Invitrogen) for two weeks. Zeocin-resistance clones were subjected to doxycycline (Clontech)-inducibility test for the expression of desired gene by western blot analysis using anti-FLAG M2 antibody (Sigma). Two independent positive clones of K-RTA with similar growth rate and expression level were pooled, designated as 293TetKR.

### Cell culture

Tet-inducible cell lines 293TetKR and ERKV [21] were maintained in DMEM supplemented with 10% Tet System Approved FBS (Clontech), 5 μg/ml blasticidin and 200 μg/ml zeocin. HEK293 and 293/rKSHV.219 were maintained in DMEM with 10% FBS (Invitrogen). Puromycin (660 ng/ml, BD Biosciences) was added to the culture media of ERKV and 293/rKSHV.219 cells for the maintenance of viral genomes. On a routine basis, all cells were maintained in a humidified 37°C incubator with 5% CO2, passaged and fed every 3 to 4 days.

### Protein purification

Immuno-purification of FLAG-tagged protein from 293Tet-inducible cell line was carried out according to two previously described procedures [22,23] with minor modifications. Specifically, one hundred million cells with 80% confluence were treated with 50 ng/ml doxycycline for 5 h and doxycycline plus 10 mM glucosamine for additional 5 h before cell harvest. Combined cell pellet (~ 1 g) was solubilized in 10 ml lysis buffer (50 mM Tris-HCl, pH 7.4, 100 mM GlcNAc, 150 mM NaCl, 1 mM EDTA, 1% Triton X-100, 1 × protease inhibitors, 0.2 mM sodium orthovanadate) at 4°C for 40 min with gentle shaking. Protein extracts were clarified by centrifugation at 12,000 × g, 4°C for 15 min. Collected protein supernatant was filtrated through a 0.45 micron filter to remove cell debris. The filtered protein supernatant was applied onto 400 μl anti-FLAG M2 affinity resin (A2220, Sigma) equilibrated with lysis buffer. The column eluate was re-loaded onto the column for two more cycles. The column was alternatively washed with one column volume of TBS (50 mM Tris-HCl, pH 7.4, 150 mM NaCl) followed by one column volume of high salt (500 mM NaCl)-TBS for three times. The column was washed with additional two column volumes of TBS buffer prior to elution. The FLAG-tagged protein was eluted from the column by TBS buffer supplemented with 100 μg/ml FLAG peptide (F3290; Sigma) and 20% glycerol. For each purification, four eluted fractions were collected and samples were immediately frozen in -70°C for further studies.

### Liquid chromatography-tandem mass spectrometry (LC-MS/MS)

The affinity purified K-RTA (~500 ng) was resolved in 8% SDS-PAGE followed by SYPRO^® ^Ruby (Invitrogen) staining. Stained region was excised and in-gel digested with trypsin (sequencing grade, Promega). The digests were first mapped by MALDI-MS analysis on an ABI 4700 Proteomics Analyzer to identify candidate *O*-GlcNAc modified K-RTA peptide, which was indicated by the presence of molecular ion signal at 204 Da above the identified tryptic peptide signal. The sample was then subjected to LC-MS/MS analysis as described previously [24] to confirm and identify the *O*-GlcNAcylated sites.

### Transfection and luciferase reporter assay

Transfection was performed in 24-well plates. HEK293 cells (2.5 × 10^5^) were seeded into each well to reach a 90% confluence the next day before transfection. Transfection was carried out using Lipofectamine™ 2000 (Invitrogen) conveying appropriate plasmids according to the manufacturer's instruction. Twenty-four h after transfection, cells were harvested for luciferase activity assay. Transfection efficiency was normalized by a co-transfected renilla luciferase reporter (phRL-TK, Promega). Firefly and renilla luciferase activities were measured by an Orion L Luminometer (Berthold Detection Systems) using Dual-Glo luciferase assay kit (Promega).

### Western blot analysis and co-immunoprecipitation

Cells were lysed in RIPA buffer (50 mM Tris-HCl, pH 8, 100 mM GlcNAc, 150 mM NaCl, 0.1% Nonidet P-40, 0.5% sodium deoxycholate, 0.1% SDS, 1 mM phenylmethylsulfonyl fluoride, 0.2 mM sodium orthovanadate, 1 × protease and 1 × phosphatase inhibitors). The protein concentration was measured spectrophotometrically at 562 nm using BCA protein assay reagent (Pierce). For each co-immunoprecipitation assay, cells were lysed in cold EBC lysis buffer (50 mM Tris-HCl, pH 7.8, 100 mM GlcNAc, 120 mM NaCl, 0.5% NP-40, 50 mM NaF, 1 mM phenylmethylsulfonyl fluoride, 0.2 mM sodium orthovanadate, and 1 × protease inhibitors). Three hundred μg protein was mixed with pre-washed anti-FLAG M2 affinity beads on a nutator mixer overnight at 4°C. After centrifugation, the resin was washed thoroughly with TBS three times followed by elution of immunocomplex in 1 × sample buffer. Cell lysates or the eluted immunocomplex were subjected to SDS-PAGE separation and analyzed by western blotting as described previously [21].

### Immunofluorescence assay

HEK293 cells at a density of 10^5^/0.4 ml/well were seeded onto a 8-well chamber slide (Nunc) coated with poly-D-Lysine (Sigma) overnight. The cells were transfected with indicated plasmids using Lipofectamine™. 2000 (Invitrogen) according to the manufacturer's instructions. Twenty-four h after transfection, cells were fixed with prechilled acetone/methanol (v/v = 1:1) at RT for 10 min and permeablied with 0.4% Triton X-100 at RT for 5 min. The slides were blocked for 30 min in blocking buffer (PBS containing 1% FBS) and incubated with anti-FLAG antibody (1: 100) in blocking buffer at RT for 2 h. The slides were washed with PBS three times for 5 min each. The slides were incubated with fluorescein isothiocyanate-conjugated anti-mouse IgG for 1 h and counterstained with DAPI (Sigma) for 5 min at RT. The slides were observed under a fluorescence microscope.

### Flow cytometric analysis

After transfection, the cells were harvested by trypsinization and resuspended in a 500 μl PBS. A total of 10,000 cells were acquired by a flow cytometer (FACSCalibur, Becton Dickinson) and analyzed using the WinMDI v2.8 software. Signals for green fluorescent protein and red fluorescent protein were detected at 488 nm and 540 nm, respectively.

### Lentivirus-based short hairpin RNAs (shRNA) knockdown of OGT

Five shRNA clones for each target protein (OGT or luciferase) were purchased from the National shRNA Core (Academia Sinica, Taipei, Taiwan). High-titer lentivirion for each shRNA clone was prepared according to the Core's instruction manual. A pilot study by using EGFP-lentivirions was conducted to optimize the target cell plating and growth, viral dose, and assay times. Subsequently, ERKV cells were seeded at a density of 6 × 10^5 ^cells/well in 6-well assay plates, incubated 24 h at 37°C, and infected with indicated lentiviral shRNA supernatant for 48 h, followed by doxycycline (50 ng/ml) induction of KSHV reactivation for 48 h before cell harvest.

### Titration of KSHV viral particles

Filtrated (0.45 μm) viral supernatant (160 μl) was incubated with 2 U DNase I (Invitrogen) at 37°C for 30 min followed by extraction of encapsidated KSHV DNA using QIAamp MinElute virus spin kit (QIAGEN). Each comparative quantitative PCR reaction was composed of 4 μl diluted viral DNA, 5 μl Power SYBR Green Master Mix (Applied Biosystems), and 1 μl primer mix (2 μM). The primers used in the present study are as follows: detection of KSHV genome, ORF9-forward (5'-CCAACATCATCCAATGCC TC-3') and ORF9-reverse (5'-GGGAAAAGTCACGGGAATG-3'). Known copy numbers of serially diluted cosmid GB11 DNA encompassing KSHV genome nt 1-35,022 (U75698) were used as standards in titrating KSHV viral particles. The reaction was conducted and detected by StepOnePlus™ Real-Time PCR system (Applied Biosystems).

## Results

### K-RTA possesses 20 potential *O*-GlcNAcylation residues

**Figure 1:**
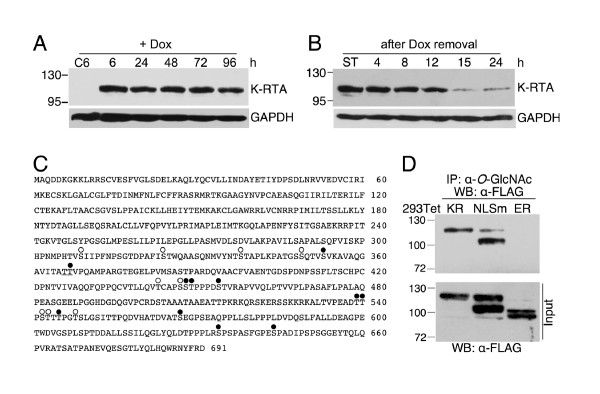
**K-RTA, but not EBV Rta, is recognized by the anti-*O*-GlcNAc antibody RL2**. (A) Expression kinetics of K-RTA in Doxycycline (Dox)-treated 293TetKR cells (50 ng/ml) for the indicated times. C6: untreated 293TetKR at 6 h. (B) Protein stability of K-RTA in 293TetKR cells. The remaining amounts of K-RTA in 24 h Dox-treated 293TetKR cells were monitored between 4 and 24 h after Dox removal. ST: starting material. (C) The 20 potential *O*-GlcNAc sites present in the primary sequence of K-RTA composed of 691 amino acids were identified using the CBS YinOYang [45] and SVM [46] prediction programs. Ser and Thr residues scored positive in both programs are denoted by circles above the amino acid symbols. Open circles indicate that residues are only *O*-GlcNAcylated; solid circles represent residues that might also be phosphorylated. Thr-366 and Thr-367 are underlined. (D) Protein extracts from 293TetKR (K-RTA), 293TetNLSm (K-RTA defective in nuclear localization signal) and 293TetER (EBV Rta) cells were immunoprecipitated by an anti-*O*-GlcNAc antibody (RL2) followed by western blot analysis using the M2-FLAG antibody. Only K-RTA and NLSm were immunoprecipitated by anti-*O*-GlcNAc antibody.

293TetKR is a recently established HEK293 cell line that displays doxycycline (Dox)-controlled, conditional expression of K-RTA. Expression of K-RTA was induced by treating 293TetKR cells with low concentration (50 ng/ml) of Dox, a physiologically neutral compound. K-RTA was detectable at 6 h and was expressed stably until 96 h in the Dox-treated 293TetKR cells by western blot analysis (Figure 1A). Removal of Dox from the 24 h treatment resulted in a near depletion of K-RTA in 15 h (Figure 1B), indicating that both synthesis and degradation of K-RTA was active and a continuous supplement of Dox is required for a sustained expression of K-RTA throughout the course of experiments. The molecular weight of K-RTA produced in 293TetKR cells (114 kDa) was apparently greater than the *in vitro*-translated species (95 kDa, not shown) and its predicted size (76 kDa), suggesting that extensive post-translational modification products might attach to K-RTA during protein maturation. K-RTA was previously hypothesized to be an abundantly phosphorylated protein [25], but the role of phosphorylation in K-RTA structure and function is unclear. Interestingly, when we inspected the 137 potential phosphorylation residues consisted of Ser, Thr and Tyr, we noticed that there are 20 Thr and Ser exhibiting a high likelihood for *O*-GlcNAcylation, and 11 of them are also potential phosphorylation sites (solid circles, Figure 1C). These predictions suggested that *O*-GlcNAc and *O*-phosphate might alternate on these Ser and Thr residues. To assess whether K-RTA is *O*-GlcNAcylated *in vivo*, protein extracts from Dox-treated 293TetKR, 293TetNLSm and 293TetER [21] were immunoprecipitated with RL2, an antibody specifically recognizing the *O*-GlcNAc epitope, followed by western blot analysis using the M2-FLAG antibody. We found that both K-RTA and its mutant defective in nuclear localization signal (NLSm), but not EBV Rta, were recognized by RL2 (Figure 1D), indicating that K-RTA is *O*-GlcNAcylated. These preliminary results prompted us to identify the authentic *in vivo O*-GlcNAcylation sites in K-RTA by mass spectrometry.

### Identification of Thr-366 and Thr-367 as the primary *in vivo O*-GlcNAcylation motif of K-RTA

**Figure 2:**
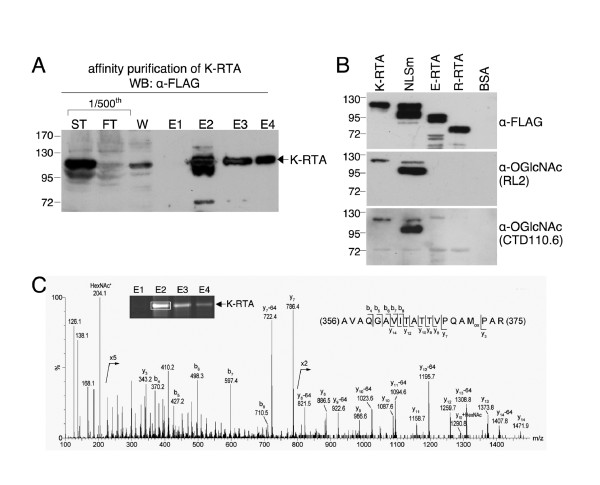
**Identification by mass spectrometry (MS) of *in vivo O*-GlcNAc sites in K-RTA**. (A) Affinity purification of FLAG-K-RTA by M2-FLAG resin. Protein extracts from 1 g (~10^8 ^cells) of Dox-treated 293TetKR cells were used as starting material. Purification was verified by western blot analysis using M2-FLAG antibody. ST, starting material; FT, flow through; W, wash; E1-E4, eluted fractions 1-4. (B) Purified K-RTA and K-RTA mutant defective in nuclear localization signal (NLSm) were readily immunoreactive to the anti-*O*-GlcNAc antibodies RL2 and CTD110.6. Aliquots of ~ 50 ng of each affinity-purified protein, including K-RTA, K-RTA NLSm (NLSm), EBV Rta (E-RTA), rhesus monkey rhadinovirus RTA (R-RTA) and 300 ng bovine serum albumin (BSA), were used to prepare one of the three identical immunoblots. The blots were respectively hybridized to anti-FLAG, RL2 and CTD110.6. Whereas the four FLAG-tagged proteins were obviously detected by anti-FLAG, only K-RTA and NLSm possess *O*-GlcNAc epitopes that were detectable by RL2 and CTD110.6. (C) MS/MS spectrum of a candidate singly *O*-GlcNAcylated tryptic peptide, AVAQGAVITATTVPQAMPAR, from the E2 fraction of the purified K-RTA (rectangle) at *m/z *724.70 (3+). The strong oxonium ions at *m/z *204 (HexNAc*), 168 and 138 confirm the presence of GlcNAc moiety but the precise site location cannot be established unambiguously because of the facile neutral loss of *O*-GlcNAc (see text).

To identify which of the 20 potential Ser or Thr amino acids are *O*-GlcNAcylated *in vivo*, FLAG-tagged K-RTA derived from Dox-treated 293TetKR cells was affinity purified using M2-FLAG resin according to two previously described procedures [22,23] (Figure 2A). A portion of the affinity-purified K-RTA was subjected to western blot analyses using two different *O*-GlcNAc antibodies, RL2 and CTD110.6. To rule out the nonspecific cross hybridization that can arise in western blot analysis, equivalent amounts of purified K-RTA, NLSm, EBV Rta, rhesus monkey rhadinovirus RTA and bovine serum albumin were analyzed in parallel. Whereas the four FLAG-tagged proteins were detected by α-FLAG, only K-RTA and K-RTA NLSm were immunoreactive to RL2 and CTD110.6 (Figure 2B), indicating the specificity of the current procedure. Further, to identify the authentic *O*-GlcNAcylation sites conclusively, purified K-RTA was subjected to LC-MS/MS analysis. As expected, a strong peak of 204.1 Da in size indicative of GlcNAc was recognized (Figure 2C, denoted by HexNAc*). The strong oxonium ions at *m/z *204, 168 and 138 confirmed the presence of a GlcNAc moiety but the precise location cannot be established unambiguously because of facile neutral loss of *O*-GlcNAc. Nonetheless, a candidate singly *O*-GlcNAcylated tryptic peptide (AVAQGAVITATTVPQAMPAR) was revealed by MS/MS spectrum and a weak signal at *m/z *1290, assigned to *O*-GlcNAcylated y_10_, suggested that the single *O*-GlcNAc is most likely attached to Thr-366 or Thr-367. Finally, the oxidized Met residue contributed to the observed -64 u loss, which is in accordance with Guan *et al. *[26] (Figure 2C). In conclusion, these results established that K-RTA is immunoreactive to two distinct *O*-GlcNAc antibodies and that an *in vivo O*-GlcNAc motif is located at Thr-366/Thr-367.

### *O*-GlcNAcylation of Thr-366 and Thr-367 is suppressive to the potency of K-RTA mediated viral promoter transactivation and lytic cycle reactivation

**Figure 3:**
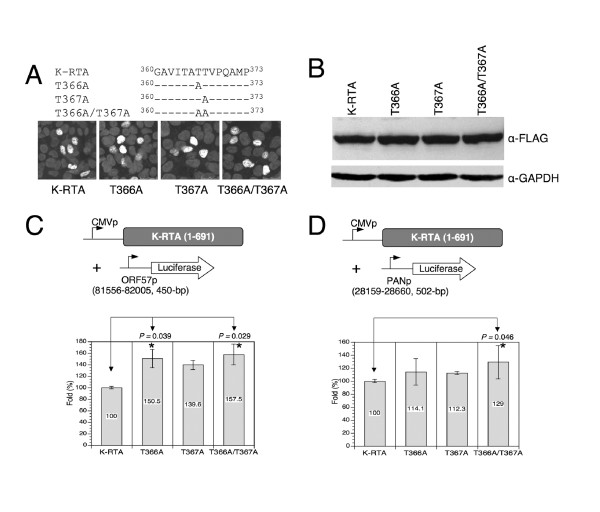
**Disruption of *O*-GlcNAcylation on T366 and T367 leads to an increase in the transactivation activity of K-RTA**. (A) Nuclear localizations of the *O*-GlcNAc mutants were similar to the wild-type K-RTA, as shown by an immunofluorescence assay using M2-FLAG antibody. (B) Western blot analysis of protein extracts from HEK293 cells transiently transfected with expression plasmids for K-RTA or *O*-GlcNAc mutants. GAPDH served as a loading control. Similar protein expression levels and migration patterns were observed between wild-type K-RTA and the three *O*-GlcNAc mutants. (C) Luciferase reporter assay of the KSHV ORF57 promoter responding to K-RTA and *O*-GlcNAc mutants in HEK293 cells. Each firefly luciferase value was normalized to an internal control plasmid, pRL-TK. The normalized value for K-RTA was set to 100%. Data are presented as the mean ± SD from triplicate transfections. * denotes *P *< 0.05 (Student's *t*-test). Six independent experiments were performed and a representative result is shown. (D) Luciferase reporter assay of the KSHV PAN promoter responding to K-RTA and *O*-GlcNAc mutants in HEK293 cells. The procedure was carried out as described in (C), except that ORF57p was replaced with PANp. Six independent experiments were performed and a representative result is shown.

To test whether *O*-GlcNAcylation of Thr-366/Thr367 is required for K-RTA's biological activity, three Thr to Ala substitution mutants were generated to mimic the unmodified status of these amino acids (Figure 3A, upper). We first confirmed that the three variants were primarily localized in the nucleus using an immunofluorescence assay (Figure 3A, lower). In addition, the protein expression levels and migration patterns of the three variants were similar to that of the wild-type K-RTA (Figure 3B). Next, we wished to compare the transactivation activities of wild-type K-RTA and the three *O*-GlcNAc site mutants. Two well-established K-RTA target promoters were used: ORF57 and PAN [27]. The results from multiple independent assays showed that although moderate, the substitutions of Thr residues at T366 and T367 constantly increased the transactivation activity of K-RTA on both KSHV early promoters (Figure 3C-D). These results implied that *O*-GlcNAcylation of Thr-366 or Thr-367 might inhibit the transactivation potency of K-RTA. This observation is reminiscent of the inhibitory effect of *O*-GlcNAcylation on cellular factor Sp1 [28,29] and C/EBPβ [30].

**Figure 4:**
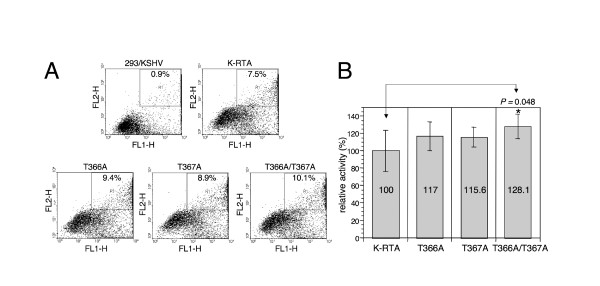
***O*-GlcNAcylation of Thr-366 and Thr-367 is involved in KSHV reactivation**. (A) Comparison by flow cytometric analysis of the potency for viral latency disruption of K-RTA and *O*-GlcNAc mutants. 293/rKSHV.219 cells transfected with K-RTA or the three *O*-GlcNAc mutants (T366A, T367A and T366A/T367A) for 72 h were analyzed on a fluorescence-activated cell sorter followed by scoring the percentage of double positive (GFP and RFP) cells in each population, as indicated in the upper right of each panel. Untreated cells (293/KSHV) served as a control. (B) A schematic chart summarizes the results derived from eight independent experiments. Data are presented as the mean ± SD with the K-RTA group set to 100%. * denotes *P *< 0.05 (Student's *t*-test).

To extend this study in cells infected with KSHV, expression plasmids of wild-type K-RTA, T366A, T367A and T366A/T367A were ectopically expressed in 293/rKSHV.219 cells, followed by scoring of the percentage of green and red fluorescence double-positive cells, an indicator of KSHV lytic reactivation [31]. At a transfection rate ~80%, lytic reactivation induced by the wild-type K-RTA was 15-28% less than that by the three mutants (Figure 4). These results are consistent with those from luciferase reporter assays (Figure 3) and together suggest that *O*-GlcNAcylation of Thr-366 or Thr-367 imposes a suppressive effect toward K-RTA during viral reactivation. It bears noting that, between Thr-366 and Thr-367, neither luciferase reporter assays (Figure 3C-D) nor lytic reactivation scoring method (Figure 4) could conclusively discern which residue is more important, suggesting that *O*-GlcNAc could be dynamically or alternatively added onto these two residues. In agreement, the double mutant, T366A/T367A, yielded more consistent and prominent effect in the aforementioned experiments.

### *O*-GlcNAcylation of Thr-366 and Thr-367 is involved in PARP1 recruitment

**Figure 5:**
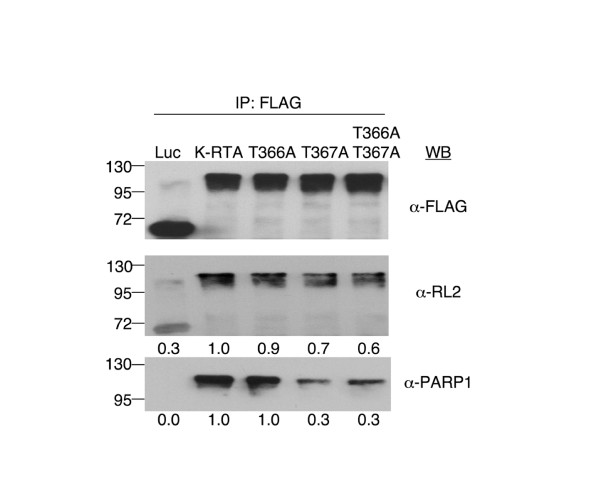
***O*-GlcNAcylation of Thr-366 and Thr-367 plays a role in PARP1 recruitment**. Protein extracts from 293T cells transiently transfected with plasmids expressing K-RTA or *O*-GlcNAc mutants for 24 h were subjected to co-immunoprecipitation (Co-IP) assays by using M2-FLAG resins. Equal aliquots of each Co-IP were resolved on SDS-PAGE followed by western blot analysis using indicated antibodies. After normalized to respective total IP proteins (α-FLAG), the relative ratios of *O*-GlcNAcylation state (α-RL2) and the amount of PARP1 recruited by K-RTA and *O*-GlcNAc mutants are denoted beneath each lane.

Among various K-RTA interacting cellular proteins, we were particularly interested in HDAC1 and PARP1 because these two molecules are known negative regulators of transcription [32,33] and have been shown to repress K-RTA's activity by Gwack and colleagues [5,34]. To this end, protein extracts of 293T cells transiently transfected with K-RTA or the three *O*-GlcNAc mutants were subjected to co-immunoprecipitation (Co-IP) assay using M2-FLAG resin. Equal aliquots of the immunocomplexes from each Co-IP were used to prepare four duplicate immunoblots, delegated for antibodies against FLAG, *O*-GlcNAc (RL2), HDAC1 and PARP1. First, the result of M2-FLAG antibody validated that the Co-IP efficiency of all FLAG-tagged protein complexes were similar (Figure 5, top panel). Next, the *O*-GlcNAcylation states of K-RTA and mutants were revealed by the *O*-GlcNAc-specific antibodies RL2 (middle panel). The degree of *O*-GlcNAcylation in the three *O*-GlcNAc mutants were less than that in the wild-type K-RTA, reinforcing that this motif is *in vivo *modified with *O*-GlcNAc. Also, the result indicates that there are additional *O*-GlcNAcylation sites in K-RTA that were missed in our mass spectrometric analysis (discussed below). Importantly, we found that while the amount of HDAC1 immunoprecipitated in each complex did not vary significantly (not shown), the amount of PARP1 in T367A and T366A/T367A was ~70% less than that in the K-RTA wild-type, suggesting that association of K-RTA with PARP1 was proportional to the state of *O*-GlcNAcylation (Figure 5, bottom panel). Taken together, these results suggested that *O*-GlcNAcylation of K-RTA facilitates its recruitment of negative regulator PARP1, which in turn dampens K-RTA activity (Figure 3, 4).

### Depletion of OGT resulted in increased KSHV reactivation

**Figure 6:**
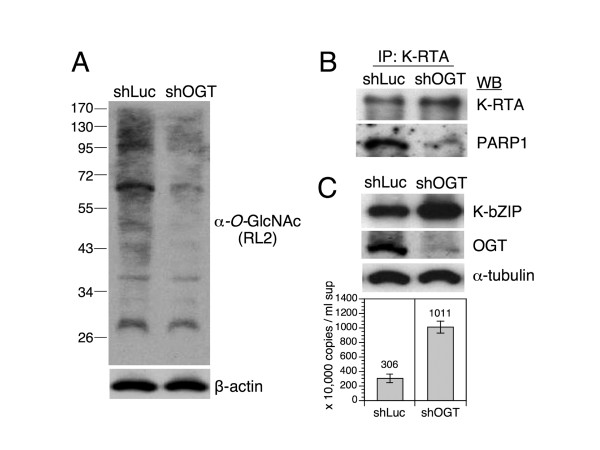
**Depletion of *O*-GlcNAc transferase (OGT) led to enhancement of KSHV reactivation**. Lentivirus-based short hairpin RNAs (shRNA) targeting luciferase (Luc), or OGT were expressed in ERKV cells for 48 h, followed by doxycycline (50 ng/ml)-induction of KSHV reactivation for additional 48 h. (A) Western blot analysis of protein extracts from shRNA-treated cells by using α-*O-*GlcNAc antibody (RL2). Compared to control shLuc, shOGT decreases the global *O*-GlcNAcylation state in ERKV cells. β-actin served as a loading control. (B) K-RTA interacts with less PARP1 in shOGT-treated ERKV cells. Protein extracts from shRNA-treated ERKV cells were subjected to co-immunoprecipitation (Co-IP) assay using α-K-RTA antibody. Equal aliquots of each Co-IP were resolved on SDS-PAGE followed by western blot analysis using indicated antibodies. (C) (upper) Western blot analysis of K-bZIP and OGT expressions in shRNA-treated ERKV cells. An enhanced expression of K-bZIP is present in the OGT knockdown cells relative to that in the control shLuc group. The expression pattern of K-bZIP is inversely correlated with the cellular OGT level. α-tubulin served as a loading control. (lower) KSHV particles released from shLuc and shOGT-treated ERKV cells. Copy numbers of DNase I-resistant, encapsidated viral DNAs in each filtrated culture supernatants were determined by comparative quantitative PCR of KSHV DNA polymerase gene (ORF9). Data are presented as means ± SD from three PCR assays in an experiment. Similar results were obtained from two independent experiments; one set of data is shown.

Given that T366A/T367A mutant is still detectable by α-*O*-GlcNAc antibody and associates with PARP1 (Figure 5), it is imprudent to use this mutant as a un-glycosylated form of K-RTA. In addition, pilot experiments showed that both wild-type K-RTA and *O*-GlcNAc variants, when provided ectopically, could induce the expression of endogenous K-RTA in a given KSHV-positive cell. This endogenous K-RTA then took over the reactivation process by which the initial effects of wild-type K-RTA and *O*-GlcNAc mutants became less distinguishable. Thereby, to explore the biological impacts of *O*-GlcNAcylation on K-RTA in KSHV-infected cells, we asked whether global decrease of *O*-GlcNAcylation by short hairpin RNAs (shRNA) knockdown of OGT would affect K-RTA mediated KSHV reactivation. To this end, shRNAs of control luciferase (shLuc) or OGT (shOGT) were delivered into ERKV cells for 48 h. ERKV is a recently established cell model by which reactivation of KSHV could be homogeneously induced by low concentration of doxycycline (Dox, 50 ng/ml) [21]. To validate the shRNA knockdown efficiency, the global *O*-GlcNAcylation state of ERKV cells receiving shLuc or shOGT were measured by western blot analysis using *O*-GlcNAc antibody RL2. As expected, shOGT noticeably decreased the overall *O*-GlcNAcylation state in ERKV cells (Figure 6A). Another *O*-GlcNAc antibody CTD110.6 also gave same result (not shown).

To evaluate the extent of KSHV lytic cycle reactivation under hypo-*O*-GlcNAcylation states, shLuc or shOGT-expressing ERKV cells were treated with Dox for 48 h, and the protein extracts of each sample were subjected to western blot analysis or immunoprecipitation assay. To examine the nature of K-RTA and PARP1 complexes in these shOGT-expressing cells, protein extracts of shLuc or shOGT-expressing cells were immunoprecipitated by α-K-RTA followed by western blot analysis using α-K-RTA and α-PARP1, respectively. As depicted in Figure 6B, despite more K-RTA was synthesized in the shOGT-expressing cells, a feature can be attributed by a positive feedback of K-RTA auto-regulation [35], the amount of PARP1 interacting with K-RTA was dramatically reduced in the shOGT cells. This data is consistent with previous findings that Thr-366/Thr-367 *O*-GlcNAc mutants associated with less PARP1 (Figure 5) and were more potent (Figure 3-4). Next, because K-bZIP is a direct downstream target of K-RTA, expression of K-bZIP was used as a surrogate marker for K-RTA mediated viral reactivation. As shown in Figure 6C, expression of K-bZIP was elevated in shOGT cells relative to that in the control group. Furthermore, we observed that K-bZIP expression was inversely correlated with the level of OGT in multiple independent experiments using different sets of shOGT RNAs (Figure 6C and data not shown), indicating that hypo-*O*-GlcNAcylation is a favorable environment for KSHV lytic cycle reactivation. This notion is further supported by a parallel increased titer of viral particles shed into the culture medium (Figure 6C). Taken together, based on these results we propose that during KSHV lytic cycle replication, more OGT will facilitate the *O*-GlcNAcylation state of K-RTA that in turn recruits more PARP1 and diminish the potency of K-RTA.

## Discussion

K-RTA is a Ser and Thr-rich protein (122 out of 691 amino acids, 17.7%) but the sites and roles of post-translational modifications (PTMs) on these residues are largely unexplored. In this study, by using mass spectrometric analysis we demonstrated that K-RTA is an *O*-GlcNAcylated protein and that Thr-366/Thr-367 is an *O*-GlcNAcylation motif. Thr to Ala mutations at this motif increased K-RTA's transactivation capability in luciferase reporter assays and KSHV reactivation in 293/rKSHV.219 cells, indicating that *O*-GlcNAcylation may impose a suppressive effect on K-RTA. This notion was further supported by increased K-bZIP expression and viral particle production in KSHV infected cells depleted with *O*-GlcNAc transferase (OGT). The inhibitory effect of *O*-GlcNAcylation on K-RTA was attributed to an increasing affinity between glycosylated K-RTA and PARP1. Noteworthy, PARylation of K-RTA by PARP1 was previously shown to negatively modulate the activity of K-RTA [5]. Thereby, our results established a link between these two PTMs in regulating K-RTA. Furthermore, given that *O*-GlcNAcylation is a dynamic process keenly responding to glucose fluctuation, we speculate that the activity of K-RTA is closely controlled by the metabolic state of the host cell. For example, Delgado et al. recently demonstrated that induction of Warburg effect in KSHV-latently infected endothelial cells is a required process for tumor cell survival [36]. As increased glucose uptake will produce more UDP-GlcNAc, in theory Warburg effect would be coupled with elevated *O*-GlcNAcylation. Thus, Warburg effect may create an environment suppressive to K-RTA's full functionality that leads to a crippled KSHV reactivation. This could explain why most KSHV remain latent in the KS biopsies.

In Figure 5, the M2-FLAG resin-precipitated T366A/T367A mutant is still reactive to α-*O*-GlcNAc antibody (RL2), indicating that Thr-366/Thr-367 is not the only site modified by *O*-GlcNAc. This partial de-*O*-GlcNAcylation in Thr-366 and Thr-367 mutants may explain why the enhancement effects in our functional assays were small, although statistically significant (Figure 3, 4). We reasoned that those unidentified *O*-GlcNAcylation sites most likely are located at larger tryptic peptides that have been excluded in our mass spectrometry (e.g., amino acids 531-633, approximately 10.6 kDa, contain 25 Ser/Thr). Alternatively, the fragile *O*-GlcNAc moieties might have been lost during protein purification. Thus, the use of other proteases such as chymotrypsin to supplement the trypsin digestion, or employing more advanced methods including "QUICK-Tag" and electron transfer dissociation mass spectrometry [37,38] should disclose additional *O*-GlcNAcylation sites in K-RTA and provide a more comprehensive biological relevance.

Suppression of transactivation activity by *O-*GlcNAcylation is best known in Sp1. An *O*-GlcNAcylated activation domain of Sp1 repelled its hydrophobic interaction with TAF110 *in vitro *[29] and removal of the *O*-GlcNAc moiety in Sp1 elicited a signal for activation [29,39]. Hyper-*O-*GlcNAcylation of Sp1 severely impaired the transactivation of p21/WAF1 promoter in HeLa cells [28], and, the LTR promoter activity of HIV-1 in T cells [40]. Other than Sp1, *O*-GlcNAcylation of C/EBPβ, YY1 and NF-κB p65 also brought suppressive transcriptional effects on their target genes [30,41,42]. Here, we showed that forced de-*O*-GlcNAcylation of Thr-366 and Thr-367 in K-RTA resulted in moderate enhancement in activity (Figure 3, 4). This motif is located in the middle of the 691 amino acids consisting of K-RTA, a region previously mapped to interact with positive regulator RBPJκ [3]. Thus, it is likely that unmodified Thr-366/Thr-367 in this region may provide a better interaction domain for RBPJκ or factors in the transcription machinery, as has been described in the case of Sp1. In addition, we found that *O*-GlcNAcylated K-RTA "attracts" more PARP1, another kind of PTM enzyme that transfers large and negatively charged polymer (PAR) onto numerous nuclear factors. Frequently, the attachment of PAR led to altered activity and function of target proteins through both steric and charge inhibition [17]. In agreement, a previous report demonstrated that PARP1 PARylated K-RTA *in vitro *and suppressed its activity in a co-expression assay [5]. Combined, we speculate that *O*-GlcNAcylated K-RTA repels more of its positive regulators, associates with more PARP1 thus being PARylated, and ultimately its full functionality is restricted. It would be of great interest to investigate whether *O*-GlcNAcylation and PARylation exert synergistic or feedback effects in modulating K-RTA.

*O*-GlcNAcylation-mediated transcriptional suppression could also take place at the chromatin level. First discovered by Yang et al., OGT was targeted by mSin3A and participated in gene silencing [13]. *Drosophila *OGT was found to be encoded by Polycomb group gene, *super sex combs *(*sxc*), and directly participated in epigenetic gene silencing on polytene chromosomes [14,15]. In mammals, murine OGT stability was regulated by polycomb repressive complex 2 and the cellular *O*-GlcNAc level was crucial to the transcription repertoire in embryonic stem cells [16]. Together, these findings provide a new perspective that OGT could be an authentic chromatin remodeller. Indeed, *O*-GlcNAcylation of H2B facilitates its subsequent monoubiquitination [43] and *in vivo O*-GlcNAcylation sites of other histone members have been revealed [44]. Intriguingly, to add one more layer of complexity, the role of PARP1 in chromatin structure modification is also increasingly evident [17]. Thereby, it is tempting to propose that modification of K-RTA by OGT and interaction of K-RTA with PARP1 may be only the secondary events. Targeting by K-RTA, OGT and PARP1 may actively modify the viral genome structures at epigenetic level before decorate and oppress K-RTA function. With this regard, future experiments are required to delineate the spatiotemporal occupancies of OGT, PARP1 and K-RTA on the viral genome and their relevance to K-RTA mediated latent-lytic switch.

We began our study by computational analysis of K-RTA primary amino acid sequences, realized that 11 of the 20 potential *O*-GlcNAcylation sites could also be *O*-phosphorylated (Figure 1C). Thr-367 is one of them. Interestingly, although our mass spectrometry did not resolve whether Thr-366 or Thr-367 is morel likely to be the real acceptor for *O*-GlcNAc, in Figure 5, substitution of Thr to Ala at Thr-367 seemed to cause a more distinct phenotype than substitution introduced at Thr-366. Thus, these hypothetical sites could serve as a blueprint for further studies in understanding alternate modifications of *O*-GlcNAcylation and *O-*phosphorylation in K-RTA.

## Conclusions

We identified Thr-366/Thr-367 in K-RTA is an *in vivo O*-GlcNAcylated motif by tandem mass spectrometric analysis. We showed that K-RTA with Thr to Ala mutations at 366 and 367 were decorated with less *O*-GlcNAc and were more potent. In agreement, forced-depletion of *O*-GlcNAc transferase (OGT) during KSHV lytic cycle replication noticeably enhanced viral lytic gene expression and viral particle production. The suppressive effect of *O*-GlcNAcylation on K-RTA was ascribed by increased capacity in PARP1 association. Noteworthy, the roles of OGT and PARP1 in chromatin modification/architecture have recently emerged; indicating that K-RTA mediated gene expression is controlled by chromatin remodeling factors.

## Competing interests

The authors declare that they have no competing interests.

## Authors' contributions

YCK, WHT and SFL conceived and designed the experiments. YCK and PWW established conditional expression cell lines and performed protein purification. ILW and SYL carried out mass spectrometric (MS) analysis and analyzed the MS data. YCK, YLC and WHT constructed *O*-GlcNAc mutants, performed luciferase reporter assay, flow cytometric analysis and immunoprecipitation. YCK and WHT participated in shRNA-mediated knockdown experiments and viral particle titration. WHT, JYC and SFL wrote the paper. All authors read and approved the final manuscript.
